# Carbon-rich materials: from polyaromatic molecules to fullerenes and other carbon allotropes

**DOI:** 10.3762/bjoc.21.62

**Published:** 2025-04-17

**Authors:** Hiroko Yamada, Yoko Yamakoshi

**Affiliations:** 1 Institute for Chemical Research, Kyoto University, Gokasho, Uji, Kyoto, 611-0011, Japan; 2 Department of Chemistry and Applied Biosciences, ETH Zürich, Zürich CH8093, Switzerland

**Keywords:** carbon allotropes, carbon-rich materials, fullerenes, polyaromatic molecules

In addition to diamond and graphite, traditional carbon allotropes in our old high-school textbook, new types of carbon allotropes, molecular carbons, were discovered in the last decades. These include fullerenes (1985) [1], carbon nanotubes (1991) [2], and graphene (2004) [3]. Due to their unique electronic and photophysical properties, research in the area of carbon-rich molecules and polyaromatic molecules became explosive in their activity and a numerous new studies and directions have emerged. Because of dramatic expansion and development of these research areas, these molecules became targets of Nobel Prizes in Chemistry (in 1996 to Carl, Kroto, and Smalley for the discovery of C_60_) and in Physics (in 2010 to Geim and Noboselov for the discovery of graphene).

Prior to the celebration of the 40th anniversary of the discovery of fullerene in 2025, we planned this thematic issue on carbon-rich materials. We highly appreciate that so many of our colleagues and friends – researchers with a variety of backgrounds working on these special molecules – agreed to contribute to this project.

The authors hail from diverse research areas, including synthetic organic chemistry, surface chemistry, computational chemistry, physics, electrochemistry, polymer chemistry, supramolecular chemistry, and biochemistry. All are working on fascinating topics associated with these exciting molecules. Furthermore, the authors come from all over the world, not only from Switzerland and Japan but also from China, Denmark, Estonia, France, Spain, Turkey, and USA.

We hope that the readers will enjoy this thematic issue and be inspired to further develop their own research in the near future.

Hiroko Yamada and Yoko Yamakoshi

Kyoto, Zurich, February 2025

## Data Availability

Data sharing is not applicable as no new data was generated or analyzed in this study.
